# Religiosity & mental health seeking behaviors among U.S. adults

**DOI:** 10.1177/00912174231187841

**Published:** 2023-06-30

**Authors:** Augustine Cassis Obeng Boateng, Joshua Sebu, Ruby Lekwauwa, Katherine C. Britt, Hayoung Oh, Benjamin Doolittle

**Affiliations:** 1 Department of Biobehavioral Sciences, School of Nursing, 16142 University of Pennsylvania, Philadelphia, PA, USA; 2 Spirituality and Health Hub, Philadelphia, PA, USA; 3 Department of Economics, 314752 University of Cape Coast, Cape Coast, Ghana; 4 School of Medicine, 5755 Yale University, New Haven, CT, USA

**Keywords:** Religious leaders, spiritual leaders, psychotherapy, mental health, adults

## Abstract

**Objectives:**

The association between religiosity and secular mental health utilization is unclear. Evidence suggests that religious and spiritual leaders (R/S leaders) may be more trusted than secular mental health therapists (SMHTs) and are often the first point of contact for individuals with mental health problems who identify as religious.

**Methods:**

Generalized equation estimate (GEE) analyses were used to examine the association between religiosity and mental health seeking behaviours in 2,107 participants using t Midlife in the United States Study (MIDUS) data collected between 1995 and 2014.

**Results:**

The final model indicated that after adjusting for covariates, higher levels of baseline religious identification and baseline spirituality (assessed in 1995) predicted an increase in visits to R/S leaders from 1995 to 2014 by a factor of 1.08 (95% CI=1.01–1.16) and 1.89 (95% CI=1.56–2.28), respectively. Higher levels of baseline religious identification reduced SMHTs visits by a factor of 0.94 (95% CI=0.90–0.98), whereas higher levels of baseline spirituality increased SMHTs visits by a factor of 1.13 (95% CI=1.00–1.27) during the same timeframe.

**Conclusion:**

Higher levels of spirituality and religious identification increased the frequency over time of seeking mental health support from R/S leaders relative to SMHTs. Individuals with mental illness may seek support from religious resources, mental health professionals, or both, underscoring the importance of collaboration between R/S leaders and SMHTs. Mental health training for R/S leaders and collaboration with SMHTs may help alleviate mental health burden, especially among those who highly value their religious and spiritual beliefs.

## Introduction

Evidence suggests that being religious is often associated with improved mental health.^1–3^ Individuals who find religion important are less likely to seek help from a secular mental health therapist (SMHT) than non-religious individuals when faced with mental health challenges.^4^ Professional organizations such as World Health Organization,^5^ American Medical Association(AMA),^6^ American Psychological Association(APA)^7^ and American Nurses Association(ANA)^8^ encourage the use of religious and spiritual dimensions in the provision of holistic care. Holistic care includes the ability of healthcare professionals to provide culturally sensitive religious and spiritual care and integrate religious and spiritual (R/S) leaders into the healthcare system.

Previous research has not explored factors that influence the inclination of individuals to seek mental health services from R/S leaders or secular mental health therapists (SMHT). The Midlife in the United States Series (MIDUS) data presents a unique opportunity to prospectively study the influence of religiosity on mental health seeking-behaviors among U.S adults.

**Definitions**: We defined SMHT as providers professionally and primarily trained to provide mental health services which may include psychologists, professional counselors, marriage therapists, licensed social workers, and psychiatrists. R/S leaders provide religious and spiritual services to individuals or groups and may include ministers, priests, rabbis or other spiritual advisors.

## Background

Religious and spiritual leaders of different traditions often play a crucial role in caring for their members’ physical, emotional, and spiritual welfare.^9^ Among communities of faith, R/S leaders often form long-lasting relationships with their members, which allows them to support each member with their health issues. Further, R/S leaders often share the same worldview and traditions as their congregants, which can be instrumental in promoting health behaviors.^9^ Religious individuals receiving care from an SMHT may have a less positive experience than non-religious individuals.^10^ Studies suggest religious teachings, shame, stigma, cost, distrust, convenience, and healthcare access are primary reasons religious people might not seek professional mental health services.^11–14^

In contrast, SMHT may not share a common perspective with religiously oriented clients, which may impede the therapeutic alliance.^15^ A study of mental health professionals’ attitudes towards spirituality and religion in treatment found that many professionals reported feeling uncomfortable discussing spiritual or religious issues with their clients and were more likely to avoid them.^16,17^ A systematic review on integrating spirituality and religion into psychotherapy found that many mental health professionals do not feel competent to address spiritual or religious issues in treatment and lack the necessary training and resources.^18,19^

Current literature on the relationship between religiosity and mental health utilization is mixed. Studies consist primarily of cross-sectional analyses, which are subject to reverse causality. The association between religiosity and mental health utilization is further complicated by intra-racial contexts, gendered lines, and barriers to accessing SMHTs. While current literature has found that non-Hispanic Blacks are less likely to use mental health services than their non-Hispanic White counterparts, there is mixed evidence of mental health utilization and religiosity. Two studies found that those with greater religiosity tended to have greater mental health utilization: one involved only Black women, and the other controlled for race.^20,21^ Another recent study found an inverse relationship between high religiosity and seeking professional mental health help among African American adults in the United States.^22^

Another study among African American women found that higher levels of religiosity were associated with lower mental health service utilization.^23^ The study found that these women were more likely to turn to religious coping strategies, such as prayer and seeking support from a religious community, rather than seeking professional mental health treatment. In addition, religiosity is associated with lower mental health service utilization among older adults with depression.^24^ The older adults were more likely to rely on religious coping strategies, such as prayer and seeking support from a religious community, rather than seeking professional mental health treatment.

However, other studies have found a positive relationship between religiosity and mental health service utilization. Specifically, one study found that higher levels of religiosity were associated with higher mental health service utilization among individuals with severe mental illness.^25^ In this study, individuals who scored high on religiosity were more likely to seek help from mental health professionals and religious or spiritual sources. Put together, these results suggest an ongoing need to investigate why individuals who find R/S important might or might not seek R/S leaders or SMHT’S when confronted with mental health challenges.

This paper elucidates the unique role of R/S leaders and secular therapists in mental health care provision in a large, longitudinal dataset. Determining why religious people may seek one of these professionals over the other may lead to targeted interventions and efforts toward fully integrating R/S leaders into the continuum of care.

**Hypothesis**: Individuals who experience a greater degree of religiosity at baseline will demonstrate a higher inclination to seek R/S leaders over SMHTs when confronted with mental health challenges.

## Methods

Data were utilized from the Midlife in the United States (MIDUS) study, supplying publicly available data on noninstitutionalized, English-speaking U.S. adults beginning in 1995.^26^ Using a random digit dialing sampling strategy, the MIDUS study aimed to examine predictors of mental and physical health in midlife adults in the U.S.^27^ and was funded by the National Institute on Aging (PO1AG20166). Approved by the institutional review board at the University of Wisconsin-Madison, the MIDUS study obtained oral informed consent from all participants. The informed consent and institutional review board approval for the MIDUS study extend to the current study using the publicly available and de-identified MIDUS data. We followed the Strengthening the Reporting of Observational Studies in Epidemiology (STROBE) reporting guidelines^28^ for cohort studies.

### Population & analytical plan

Utilizing this ongoing national study on aging and health spanning 20 years, we used data from the original MIDUS (MIDUS 1) in 1995-1996 and in the longitudinal follow-up of waves 2 (MIDUS 2) in 2004-2006 and 3 (MIDUS 3) in 2013-2014. Data were collected in 1995-1996 from U.S. adults aged 25–74 years (n = 7108) for the first wave, in 2004 to 2006 from adults aged 35–86 years (n = 4963; 75% of original respondents in Wave 1) in the second wave, and in 2013-2014 from adults aged 42–92 years (n = 3294; included 77% of Wave 2 respondents) in the third wave.

**Figure 1.**
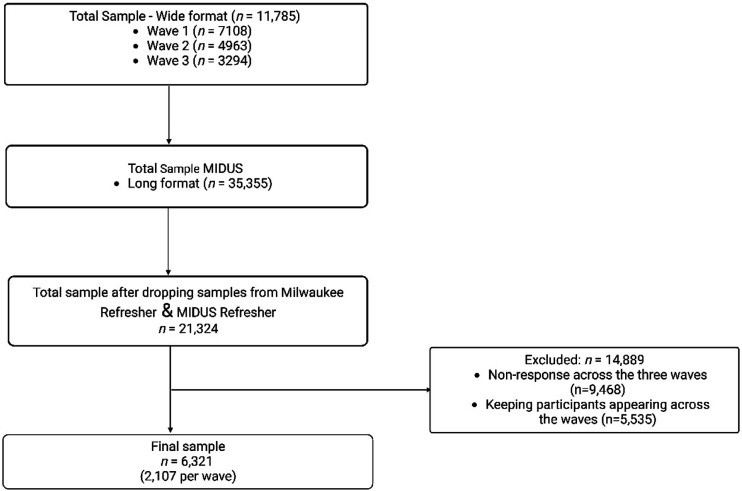
Flow diagram of sample derivation.

The study used a balanced panel dataset of 2107 respondents with complete data for all variables from waves 1-3 (1995-2014). The data was collected through phone interviews and self-administered questionnaires. The initial dataset of 11,785 samples was reshaped into a long format, producing an overall number of 35,355. We removed 21,324 samples belonging to the Milwaukee, MIDUS Refresher, and Milwaukee Refreshers because of incomplete data for all three waves. After deleting missing samples and respondents who did not appear in all three waves (see Figure 1), a final sample of 2107 was achieved for each wave representing 6321 responses for all three waves.

### Measures

Religiosity was ascertained through two self-administered spirituality and religious identification questionnaires. The spirituality scale involved two items: “How spiritual are you?” and “How important is spirituality in your life?” The religious identification scale involved six items with the following questions: “How religious are you?” “How important is religion in your life?” “How important is it for you -- or would it be if you had children now -- to send your children for religious or spiritual services or instruction?” “How closely do you identify with being a member of your religious group?” “How much do you prefer to be with other people who are the same religion as you?” “How important do you think it is for people of your religion to marry other people who are the same religion?” “How religious are you?” with four answer choices from *not at all* to *very*. The two scales are scored by summing the individual items. The scores are recoded so that higher scores reflect higher levels of spirituality or religious identification.

Chronic health conditions were measured by yes/no responses to self-reported health conditions experienced or treated in the last 12 months: anxiety or depression, diabetes, asthma, bone or joint disease (eg, arthritis, rheumatism), stroke, tuberculosis, ulcer, and neurological disorder (multiple sclerosis, epilepsy, or other neurological disorder). Chronic health conditions were grouped into two categories: mental illness (presence of anxiety and depression, or one of the two) and physical multimorbidity (diabetes, asthma, bone or joint disease, stroke, tuberculosis, ulcer, and neurological disorder). Participants with two or more self-reported physical health conditions were captured as having physical multimorbidity.

Seeking mental health services was measured by asking how many times a participant saw each of the following professionals: R/S leaders (minister, priest, rabbi, or other spiritual advisors) and SMHT (psychologists, professional counselors, marriage therapist, licensed social workers, and psychiatrist), in the last 12 months about an emotional or mental health problem. Mental health services included individual and group sessions regarding the respondent’s own issues. The original questionnaire grouped physicians (doctors) as part of secular mental health professionals. However, doctors do not meet our definition of SMHT. As such, they were excluded from the analysis.

We included potential confounders that could impact covariates which included Activities of Daily Living (ADLs), presence of insurance, age [(young adults, 25+), (middle age, 45+), (older age, 64+)], sex, education (grade school, high school or GED, college, graduate school), money (more money than you need, just enough money, not enough money), marital status, and race.

### Statistical analysis

Panel data modeling with Generalized Estimating Equation (GEE) using the negative binomial distribution^29^ was performed to examine the association between religious identification, spirituality and seeking mental health services in 1995-1996, 2004-2006, and 2013-2014 (MIDUS 1, 2, and 3) adjusting for covariates in 2107 participants. The generalized estimating equation (GEE) considers the possibility of correlation between single participant characteristics over time. Baseline exposure variables (wave 1) were used to predict outcome variables across the three waves. Data were longitudinally analyzed over three-time points using STATA 17. Two-sided statistical tests were conducted using a significance level of *p* < .05 and 95% confidence intervals (C.I.s).

## Results

Among the respondents, 56% identified as female, 74% were married in MIDUS 1, and fell to 68% in MIDUS 3, whereas nonwhites comprised 5% of the sample. The average age of respondents was 46, 55, and 64 years for MIDUS 1, 2, and 3, respectively, indicating a progressive aging over time. There was very little change in respondents’ educational attainment over the years. About 77% of respondents had just enough money to more money than needed in MIDUS 1 but it increased to 83% in MIDUS 2 and fell marginally to 82% in MIDUS 3 (Table 1). There is a steady increase (5%, 8%, and 11%, respectively) in respondents who suffer long-term physical multimorbidity. However, this is the reverse (11%, 9%, and 8%, respectively) for participants who reported having mental illness. Adoption of one form of health insurance increased from 72% in MIDUS 1–82% in MIDUS 3.

**Table 1.** Descriptive Statistics of Variables.

Variable	MIDUS 1				MIDUS 2				MIDUS 3			
	Mean	Std. Dev.	Min	Max	Mean	Std. Dev.	Min	Max	Mean	Std. Dev.	Min	Max
R/S leader bisit	.20	1.62	0	42	.19	1.56	0	40	.13	1.41	0	52
SMHT bisit	1.02	4.81	0	70	.71	5.13	0	144	.66	4.05	0	72
Religious identification	16.62	4.74	4	24	16.54	4.89	2	24	16.38	5.08	2	24
Spirituality	6.15	1.64	2	8	6.42	1.60	1	8	6.42	1.65	1	8
Mental health illness	.11	.32	0	1	.09	.28	0	1	.08	.28	0	1
Physical multimorbidity	.05	.22	0	1	.08	.28	0	1	.11	.31	0	1
ADL	.04	.24	0	2	.11	.36	0	2	.20	.49	0	2
Insurance	.72	.45	0	1	.75	.43	0	1	.82	.38	0	1
Age												
Young adult	.47	.50	0	1	.21	.41	0	1	.02	.14	0	1
Middle age	.49	.50	0	1	.62	.49	0	1	.56	.50	0	1
Old age	.04	.20	0	1	.17	.38	0	1	.42	.49	0	1
Male	.44	.50	0	1	.44	.50	0	1	.44	.50	0	1
Education												
Grade school	.01	.08	0	1	.01	.09	0	1	.01	.09	0	1
High school/GED	.28	.45	0	1	.27	.44	0	1	.27	.44	0	1
College	.52	.50	0	1	.50	.50	0	1	.51	.50	0	1
Graduate school	.19	.39	0	1	.22	.42	0	1	.22	.41	0	1
Money												
More money than you need	.20	.40	0	1	.30	.46	0	1	.27	.44	0	1
Just enough money	.57	.50	0	1	.53	.50	0	1	.55	.50	0	1
Not enough money	.23	.42	0	1	.17	.38	0	1	.18	.39	0	1
Married	.74	.44	0	1	.74	.44	0	1	.68	.47	0	1
Nonwhite	.05	.22	0	1	.05	.22	0	1	.05	.22	0	1
N (sample size)	2107				2107				2107			

*Note.* Mean values are interpreted as proportions for the binary variables and could be multiplied by 100 for percentages.

R/S, religious/spiritual; ADL, activities of daily living; SMHT, secular mental health therapist.

The results of the association between baseline religious identification and seeking mental health care controlling for covariates from 1995-2014 appear in Model 1 (Table 2). Higher levels of baseline religious identification increased the incidence of visits to the R/S leaders for mental health care by a factor of 1.19 [95% CI, 1.12, 1.27]. However, higher levels of baseline religious identification reduces the incidence of visits to SMHT by a factor of .96 [95% CI, .93, .99]. Mental illness increases the incidence of visits to R/S leaders and SMHT decades later by a factor of 3.62 [95% CI, 2.17, 6.04] and 6.84 [95% CI, 4.88, 9.58], respectively. Participants in the middle age and old age groups had reduced incidence of visits by a factor of .45 [95% CI, .30, .67] and 0.51 [95% CI, .36, .73] to R/S leaders, and .23 [95% CI, .11, .49] and .27 [95% CI, .15, .49] to SMHT respectively as compared to young age. Being married reduces the incidence of seeking mental health care compared to non-married participants to both R/S leaders and SMHT (Table 2).

**Table 2.** Model 1: Multivariate Analysis of the Relationship Between Religious Identification (Wave 1) and Mental Health Visits (Wave 1, 2, 3) Controlling for Covariates.

	R/S Leader Visit	SMHT Visit
Religious identification	1.19^***^	.96^*^
	[1.12, 1.27]	[.93, .99]
Mental illness	3.62^***^	6.84^***^
	[2.17, 6.04]	[4.88, 9.58]
Physical multimorbidity	1.49	1.16
	[.56, 3.98]	[.63, 2.11]
ADL	1.31	.68^*^
	[.87, 1.99]	[.47, .98]
Health insurance	.83	1.02
	[.53, 1.32]	[.74, 1.41]
Age: (Ref: Young adult)		
Middle age	.45^***^	.51^***^
	[.30, .67]	[.36, .73]
Old age	.23^***^	.27^***^
	[.11, .49]	[.15, .49]
Male: (Ref: Female)	.90	.76
	[.55, 1.50]	[.54, 1.08]
Education: (Ref: Grade school)		
High School/GED	.35	.31
	[.07, 1.66]	[.07, 1.30]
College	.54	.42
	[.12, 2.34]	[.10, 1.70]
Graduate School	.63	.70
	[.14, 2.86]	[.17, 2.87]
Money: (More money than you need)		
Just enough money	1.14	.70
	[.67, 1.94]	[.47, 1.05]
Not enough money	1.48	1.14
	[.82, 2.67]	[.71, 1.83]
Married: (Ref: Not married)	.53^*^	.62^**^
	[.32, .88]	[.44, .89]
Nonwhite: (Ref: White)	.62	.75
	[.32, 1.21]	[.41, 1.37]
Observations	6321	6321
Chi2	233.39	304.50

Exponentiated coefficients; 95% confidence intervals in brackets; ^*^p < .05, ^**^p < .01, ^***^p < .001.

R/S, religious/spiritual; ADL, activities of daily living; SMHT, secular mental health therapist.

The results of the association between baseline spirituality and seeking mental health care adjusting for covariates from 1995-2014 appear in Model 2 (Table 3). Higher levels of baseline spirituality increases the incidence of visits to the R/S leaders for mental health care by a factor of 2.16 [95% CI, 1.81, 2.57]; however, no significance was recorded for visits to SMHTs. Further, mental illness increases the incidence of visits to both R/S leaders and SMHT by a factor of 3.13 [95% CI, 1.92, 5.10] and 6.81 [95% CI, 4.84, 9.59], respectively.

**Table 3.** Model 2: Multivariate Analysis of the Relationship Between Spirituality (Wave 1) and Mental Health Visits (Wave 1, 2, 3) Controlling for Covariates.

	R/S Leader Visit	SMHT Visit
Spirituality	2.16^***^	1.02
	[1.81, 2.57]	[.93, 1.12]
Mental illness	3.13^***^	6.81^***^
	[1.92, 5.10]	[4.84, 9.59]
Physical multimorbidity	1.61	1.18
	[.58, 4.45]	[.64, 2.19]
ADL	1.35	.67^*^
	[.86, 2.12]	[.46, .97]
Health insurance	.96	1.01
	[.61, 1.52]	[.73, 1.40]
Age: (Ref: Young adult)		
Middle age	.43^***^	.50^***^
	[.28, .66]	[.36, .72]
Old age	.22^***^	.26^***^
	[.11, .47]	[.15, .47]
Male: (Ref: Female)	.99	.81
	[.60, 1.63]	[.58, 1.14]
Education: (Ref: Grade school)		
High school/GED	.20	.32
	[.04, 1.02]	[.08, 1.35]
College	.26	.46
	[.06, 1.25]	[.12, 1.83]
Graduate school	.30	.76
	[.06, 1.46]	[.19, 3.06]
Money: (More money than you need)		
Just enough money	1.14	.70
	[.67, 1.92]	[.46, 1.05]
Not enough money	1.57	1.15
	[.88, 2.78]	[.72, 1.85]
Married: (Ref: Not married)	.59^*^	.60^**^
	[.36, .95]	[.41, .85]
Nonwhite: (Ref: White)	.56	.71
	[.31, 1.01]	[.38, 1.31]
Observations	6321	6321
Chi2	357.52	300.31

Exponentiated coefficients; 95% confidence intervals in brackets; ^*^p < .05, ^**^p < .01, ^***^p < .001.

R/S, religious/spiritual; ADL, activities of daily living; SMHT, secular mental health therapist.

The final model (Table 4) included baseline spirituality and religious identification. We found that both measures have a positive and significant relationship with R/S leader visits, but the effect is decreased. Higher levels of baseline religious identification increases visits to R/S leaders by a factor of 1.08 [95% CI, 1.01, 1.16], while higher levels of baseline spirituality increases visits by a factor of 1.89 [95% CI, 1.56, 2.28]. Conversely, higher levels of baseline religious identification still reduces SMHT visits by a factor of .94 [.90, .98], while higher levels of baseline spirituality increases SMHT visits by a factor of 1.13 [95% CI, 1.00, 1.27]. We found no relationship between physical multimorbidity and preference for R/S leaders or SMHTs in all three models.

**Table 4.** Final Model: Multivariate Analysis of the Relationship Between Religious Identification (Wave 1), Spirituality (Wave 1) and Mental Health Visits (Wave 1, 2, 3) Controlling for Covariates.

	R/S Leader Visit	SMHT Visit
Religious identification	1.08^*^	.94^**^
	[1.01, 1.16]	[.90, .98]
Spirituality	1.89^***^	1.13^*^
	[1.56, 2.28]	[1.00, 1.27]
Mental illness	3.43^***^	6.89^***^
	[2.08, 5.66]	[4.93, 9.64]
Physical multimorbidity	1.65	1.13
	[.60, 4.55]	[.62, 2.04]
ADL	1.33	.68^*^
	[.86, 2.07]	[.47, .99]
Health insurance	.92	1.03
	[.58, 1.44]	[.75, 1.41]
Age: (Ref: Young adult)		
Middle age	.43^***^	.52^***^
	[.28, .65]	[.36, .73]
Old age	.22^***^	.27^***^
	[.10, .48]	[.15, .49]
Male	1.00	.80
	[.61, 1.63]	[.57, 1.12]
Education: (Ref: Grade School)		
High school/GED	.24	.29
	[.05, 1.22]	[.07, 1.21]
College	.33	.39
	[.07, 1.54]	[.10, 1.55]
Graduate school	.37	.63
	[.08, 1.79]	[.15, 2.58]
Money: (More money than you need)		
Just enough money	1.14	.70
	[.68, 1.92]	[.47, 1.05]
Not enough money	1.59	1.17
	[.89, 2.84]	[.73, 1.86]
Married: (Ref: Not married)	.56^*^	.64^*^
	[.34, .92]	[.45, .90]
Nonwhite: (Ref: White)	.54	.72
	[.29, 1.00]	[.40, 1.30]
Observations	6321	6321
Chi2	371.30	317.30

Exponentiated coefficients; 95% confidence intervals in brackets; ^*^p < .05, ^**^p < .01, ^***^p < .001.

R/S, religious/spiritual; ADL, activities of daily living; SMHT, secular mental health therapist.

## Discussion

We examined the unique role of R/S leaders and SMHT in mental health care provision among participants who enrolled in the MIDUS study between 1995-2014 and considered themselves religious. Findings suggest an association between higher levels of baseline spirituality and seeking mental health services from R/S leaders and SMHTs. Contrary to our hypothesis, mental illness and spirituality significantly increases the odds of seeking mental health services from R/S leaders and SMHTs. However, marital status and age reduce the odds of seeking mental health services from R/S leaders and SMHTs. We did not find an interaction effect between religious identification, spirituality, and mental illness on seeking mental health services from the two groups of providers.

These results are consistent with prior research regarding the association between religiosity and mental health service utilization in individuals with multiple health conditions. Findings from Pickard^30^ and Harris et al3^31^ indicate that religiosity may affect mental health service utilization depending on an individual's level of distress (ie, moderate vs. severe mental illness). As mental illness is known to increase heightened levels of perceived stress and psychological strain, it may lead to an increase in seeking mental health care.

The finding that mental illness significantly increases the odds of seeking mental health services from R/S leaders and SMHTs may offer one of many plausible explanations for the negative relationship observed between religious people and seeking secular mental health care in previous studies.^12^ In part because early studies of mental health utilization among religious people have focused on using secular mental health care providers rather than R/S providers to support coping and reductions in psychological distress.^32–34^ However, for religious individuals, the services provided by R/S leaders and SMHTs may be intertwined and inseparable.

In addition, this finding supports the growing need for holistic care within the healthcare system in America. Individuals may adopt a holistic approach to their healthcare, recognizing that physical, mental, and spiritual aspects of their health are interconnected, and that addressing mental health challenges requires attention to all dimensions of their well-being, including seeking support from both R/S leaders and SMHTs.

Religious adults with mental illness may seek mental health support from R/S leaders in conjunction with SMHTs for various reasons. Religious older adults find solace and comfort in their faith when facing serious illness. A review of the literature on religion and health found that religious involvement is often associated with positive health outcomes, including better physical and mental health, higher levels of well-being, and lower mortality rates.^35^ In addition, a study of terminally ill patients found that more religious people reported lower levels of distress and better coping abilities.^36^ These findings suggest that religiosity and religious involvement may be essential means by which those facing serious mental illness may regulate their emotional experiences in the context of the stress of their disease process. In this setting, R/S leaders may be an essential source of support and guidance during increased illness-related stress.

Individuals who consider themselves religious or spiritual may also believe that mental health issues are best addressed through spiritual means, such as prayer, meditation, and other spiritual practices. Perhaps, one of many reasons why the odds of seeking out R/S leaders are significantly higher than that of SMHTs. In addition, higher levels of baseline religious identification reduced the odds of seeking out SMHTs compared to R/S leaders. For example, a study of religious coping strategies found that religious individuals who used spiritual coping strategies reported lower levels of depression and anxiety.^37^ In addition, a review on the effectiveness of spiritual interventions for mental health found that spiritual interventions can effectively reduce symptoms of anxiety, depression, and substance abuse.^38^ These findings suggest that religious adults with mental illness may use R/S practices as an effective means to reduce levels of mental distress and may seek out R/S leaders more to support their use of religion to this end.

Finally, people who age with their religious/spiritual beliefs may feel more comfortable discussing their mental health challenges with someone who shares their faith and understands their belief system. Evidence from Israel^10^ and the United Kingdom^39^ suggests religious patients emphasized the significance of seeking care from a provider who shares their religiosity and affiliation and the necessity of having providers who are aware of their cultural background.

We acknowledge that while seeking help from R/S leaders may benefit some individuals, it is not a substitute for professional mental health treatment. Therefore, support from qualified SMHTs and services provided by R/S leaders may be warranted when facing mental health challenges. Our findings also underscore the potential role of R/S leaders as co-participants in caring for a patient’s mental health. For example, our results show that people with multiple health conditions seek out R/S leaders and SMHTs. This suggests that supporting R/S leaders in understanding how to identify and support mental health concerns and offering ready pathways for referrals to trusted SMHTs may be an essential resource for congregants.

To enhance collaboration between R/S leaders and SMHTs, both professions should recognize and appreciate the expertise, perspectives, and resources each group offers. This promotes holistic care that addresses each individual’s spiritual, emotional, and psychological well-being.

## Limitations

Several limitations should be considered. First, our sample consisted of primarily Christian participants, so these findings may not be generalized across other non-Christian religious traditions. Focused studies among various minority religious traditions in the United States, such as those who identify as Muslim or Jewish, are essential since these communities have different belief systems and may also have different perspectives on psychological distress and the role of R/S in its management. Second, R/S expression varies greatly within any particular religious tradition. The quality and intensity of R/S belief may greatly impact mental health-seeking behaviors. For example, conservative Christians are more likely to seek care from R/S leaders.^30^ Similarly, one’s understanding of God as benevolent or cruel is associated with mental health and help-seeking behaviors.^40,41^ The nuance of individual belief is difficult to capture in large, quantitative studies. Qualitative studies that explore belief systems and impressions of God are helpful complementary studies to explore these findings further. Third, this study was not designed to study the efficacy of R/S and secular mental health therapists. However, incorporating R/S interventions into secular mental health therapy in a coordinated fashion with R/S leaders may be helpful for certain patients.^42^

Furthermore, the findings of this study should be interpreted in the context of the questionnaire used. The number of times an individual sees a healthcare provider may not always be an accurate indicator of service utilization or effective treatment for several reasons. The frequency of visits may vary depending on the type and severity of the treated condition. Some mental health conditions require regular visits, while others only necessitate occasional check-ups. Therefore, a higher or lower number of visits may not reflect effective treatment. Recent advancements in technology and telehealth may minimize the number of physical visits.^43^ Finally, effective mental health treatment involves a therapeutic alliance between the provider and patient irrespective of the number of visits. As such, the quality and effectiveness of treatment cannot be solely determined by the number of visits.

Finally, the total sample size of our study (2107) accounted for only 29.6% of the initial respondents in wave 1. While this did not impact the statistical power or sample size, it presents challenges in generalizing our findings. The participants lost to follow-up across the three time points might possess distinct characteristics that differ from participants who remained in the study. For example, variations in their religiosity or inclination to seek R/S leaders for mental health challenges may hinder an accurate representation of the broader population and a comprehensive understanding of the relationship being examined.

## Implications

In this prospective study, participants with mental illness were more likely to seek mental health care from R/S leaders and SMHTs. This suggests that more vulnerable populations could benefit from integrative healthcare employing salient cultural resources and secular psychotherapy. Patients with higher levels of religious identification and spirituality are more likely to engage with their R/S leaders rather than mental health professionals, highlighting the significant importance of R/S leaders in the mental health of their congregants. This association is particularly notable as clergy have broad leadership and pastoral care responsibilities and may not have adequate time or training to address the most vulnerable.

Further, clergy often function within a single religious community and lack the resource network of social workers and medical professionals to provide comprehensive care. Improving R/S leaders’ training in identifying and referring the most fragile clients and connecting R/S leaders with appropriate support services is critical.

## Conclusion

People with higher levels of baseline spirituality and religious identification are more likely to seek mental health support from R/S leaders relative to SMHTs decades later. As mental health declines, religion becomes more important, and R/S leaders can play an important role in helping those with mental health challenges cope with their health. With proper training and collaboration, both R/S leaders and SMHTs can work in tandem in reducing mental health disparities in the U.S. Specific research is needed to inform whether adults who sought mental health services from R/S leaders or SMHTs had better health outcomes and why that might be.
